# Sarcoidosis presenting with glazy mucoid sputum and dyspnea: a case report

**DOI:** 10.1186/s13256-021-02809-2

**Published:** 2021-05-11

**Authors:** Wud Al-Kailany, Wim Timens, Ben Venmans, Gonda de Jonge, Tjip S. van der Werf

**Affiliations:** 1 Department of Pulmonary Diseases and Tuberculosis, University Medical Center Groningen, University of Groningen, AA11, PO Box 30001, 9700 RB Groningen, The Netherlands; 2 Department of Pathology, University Medical Center Groningen, University of Groningen, Groningen, The Netherlands; 3 Department of Radiology, University Medical Center Groningen, University of Groningen, Groningen, The Netherlands; 4 Department of Internal Medicine, Infectious Diseases, University Medical Center Groningen, University of Groningen, Groningen, The Netherlands; 5 The Medical Center Leeuwarden, Department of Pulmonary Diseases, Leeuwarden, The Netherlands; 6 Ziekenhuis Amstelland, Laan van de Helende Meesters 8, 1186 AM Amstelveen, The Netherlands

**Keywords:** Sarcoidosis, Mucoid sputum, Anti-tumor necrosis factor alpha

## Abstract

**Background:**

Patients with pulmonary sarcoidosis commonly present with a dry cough; a productive cough suggests a complicating airway infection or an alternative diagnosis such as tuberculosis or bronchiectasis.

**Case presentation:**

A 36-year-old European (Frisian) woman recently diagnosed with pulmonary sarcoidosis presented with debilitating exertional dyspnea and cough productive of glazy mucoid sputum. Several different attempts including video-assisted thoracoscopic biopsies failed to reach a second or alternative diagnosis including an infectious, autoimmune or collagen-vascular condition. She responded to steroids but with poor tolerance to this treatment, which could not be tapered. After she was started on anti-tumor necrosis factor alpha (TNF-α) therapy with infliximab, 200 mg at three-monthly intervals, she has been fine for well over a decade.

**Conclusions:**

In this patient with sarcoidosis who had a productive cough accompanied by fever, an extensive workup and prolonged follow-up, an alternative or second diagnosis could be ruled out; we therefore conclude that this highly unusual presentation is part of the clinical spectrum of sarcoidosis.

## Introduction

Sarcoidosis is a chronic inflammatory condition characterized by granulomatous inflammation of unknown origin [1]. Both pulmonary and extrapulmonary symptoms and signs may be present as clinically recognizable syndromic patterns, but unusual presentations may be challenging [2]. A dry cough is common [3], but a productive cough suggests an alternative diagnosis.

We present the case history of a patient that meets the classical radiographic and histopathological pattern of sarcoidosis, complicated by a cough productive of glazy, mucoid sputum. Based on an extensive diagnostic workup, combined with a persistent beneficial response to anti-inflammatory treatment alone, without any antimicrobial or other treatment modalities, we propose that this unusual, unique presentation should be considered part of the spectrum of the symptomatology of sarcoidosis.

## Case presentation

**Fig. 1:**
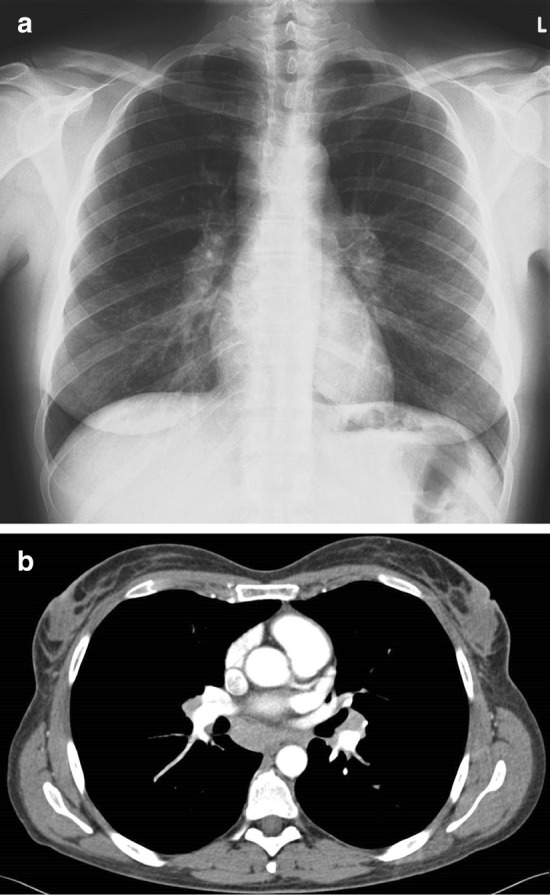
**a** Chest radiograph showing infiltrative minimal abnormalities in the left upper field, and bi-hilar and mediastinal masses suggestive of sarcoidosis. **b** Computed tomography scan image confirming bi-hilar lymph node enlargement

**Fig. 2:**
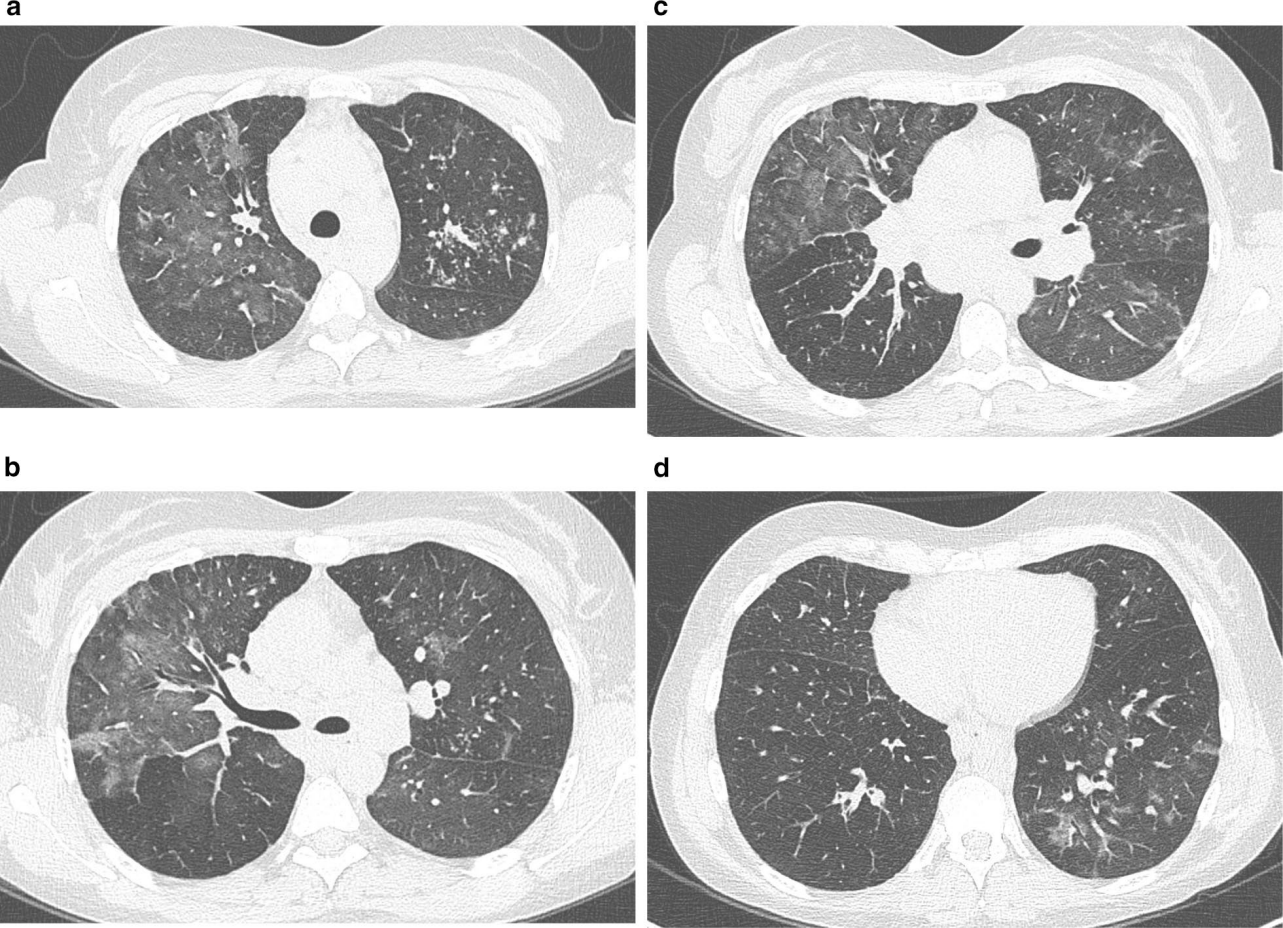
**a**–**d** High-resolution computed tomography scan image showing ground-glass attenuation in right upper and middle lobe, and in the left upper lobe, with a few scattered nodular lesions

In 2004, a then 36-year-old European (Frisian) woman was referred because of fever, dyspnea and a cough productive of shiny glazy mucoid sputum, accompanied by arthralgias. She was a lifetime non-smoker, had worked as a part-time teacher for hair dressing students but had no inhalational exposure to organic dust. Chest auscultation revealed coarse and fine crackles especially over the right anterior lung field, and sporadic scattered wheezes. Laboratory findings showed only mildly elevated C-reactive protein; no blood eosinophilia was found. At the first manifestation of her chest symptoms, sputum cultures grew *Staphylococcus aureus*, *Acinetobacter baumannii*, *Calcoaceticus complex* and *Haemophilus influenzae*; she had received targeted antimicrobial treatment without relief of her symptoms. Her chest X-ray and computed tomography (CT) scan showed mediastinal and bi-hilar lymphadenopathy, mainly suggestive for sarcoidosis stage 1 (Fig. 1a, b). Bronchoscopic lung lavage showed lymphocytic inflammation; bronchoscopic biopsies revealed loose granulomas. Cultures from blood, sputum and lavage fluid did not show bacterial, fungal or mycobacterial pathogens. Her chest symptoms improved after starting 30 mg of prednisolone daily, although during steroid therapy she did not feel well and could hardly sleep. Two years later, after several attempts to wean her from steroids, all of which were followed by recurrence of all symptoms including fever and productive cough, she was referred to our University Medical Center. She had then tapered steroids to 15 mg daily, with osteoporosis prophylaxis. No diagnosis other than the initial diagnosis of sarcoidosis could be made; attempts to further taper steroids failed. In two other specialized centers for sarcoidosis in the Netherlands, her clinical presentation with productive cough had been considered incompatible with the diagnosis of sarcoidosis; therefore, an infectious condition was suspected but not confirmed. In an attempt to further taper prednisolone, she was started on inhaled budesonide combined with salmeterol. We introduced methotrexate (15 mg weekly) as a steroid-sparing regimen, as suggested in Dutch national guidelines at the time. She however experienced gastrointestinal side effects, while steroids could not be tapered during methotrexate treatment, which we therefore subsequently stopped. When she experienced a subsequent exacerbation of disease activity in 2006, with fever, dyspnea and cough productive of the same whitish glazy material, she was admitted to the hospital. Her past medical history revealed no new information; she had had two uncomplicated pregnancies with two healthy children; the family history was negative for sarcoidosis, tuberculosis and bronchiectasis. Apart from the obstetric care, she had never received medical or socio-psychological care, or been prescribed medications other than those for her current chest symptoms. She had only traveled to Mediterranean countries for family holidays, with no exposure to respiratory infections, fumes, or organic or inorganic dusts. She was a lifetime nonsmoker, and no one in the family smoked indoors. Her alcohol intake was limited to an occasional glass of wine on weekends; there was no illicit drug use. Because of her chest symptoms, she had given up her work; she denied any earlier change in her condition when she had occasionally tried to resume work. Her medications included inhaled budesonide 250 µg and salmeterol 50 µg inhaled twice daily, oral prednisolone 5 mg daily, calcium 500 mg, and actonel 35 mg/week. On examination, she was in distress: blood pressure 95/55 mmHg; pulse 99 beats per minute; pulse oxygen saturation 98%, respiration 25 breaths per minute. Temperature was 38.6°C. No skin or eye abnormalities were detected—in particular no evidence of erythema nodosum, induration of scars or iridocyclitis was noted, and no enlarged lymph nodes were found on palpation. There was an expiratory wheeze, no crackles; heart sounds were normal. Abdomen and extremities were normal. Routine lab exams showed increased C-reactive protein to 110 mg/L; white blood cell count 19.4 ×10^9^/L, deemed as consistent with steroid use; hemoglobin 6.8 mmol/L (mild anemia); all other blood chemistry results including liver enzymes, electrolytes and renal function parameters were in the normal range. Arterial blood gas analysis showed pH 7.55, partial pressure of carbon dioxide 3.3 kPa, partial pressure of oxygen 9.9 kPa, oxygen saturation 97%, and bicarbonate ions 21 mmol/L; gas exchange for oxygen was impaired: the calculated alveolar-arterial oxygen difference was 6 kPa (normal range, 1–2 kPa). Blood and sputum cultures and a multiplex polymerase chain reaction (PCR) test for common respiratory pathogens (influenza, respiratory syncytial virus, coronaviruses, rhinoviruses, *Human metapneumovirus* and *Mycoplasma pneumoniae*) and urine tests for *Legionella pneumophila* type 1 and *S. pneumoniae* were all negative: no bacterial, fungal, parasitic or viral pathogens were identified. Pulmonary function testing showed mild restriction. High-resolution CT scanning showed, besides mediastinal and bi-hilar lymphadenopathy, ground-glass attenuation predominantly in the upper lobes without bronchiectasis (Fig. 2a–d).

**Fig. 3:**
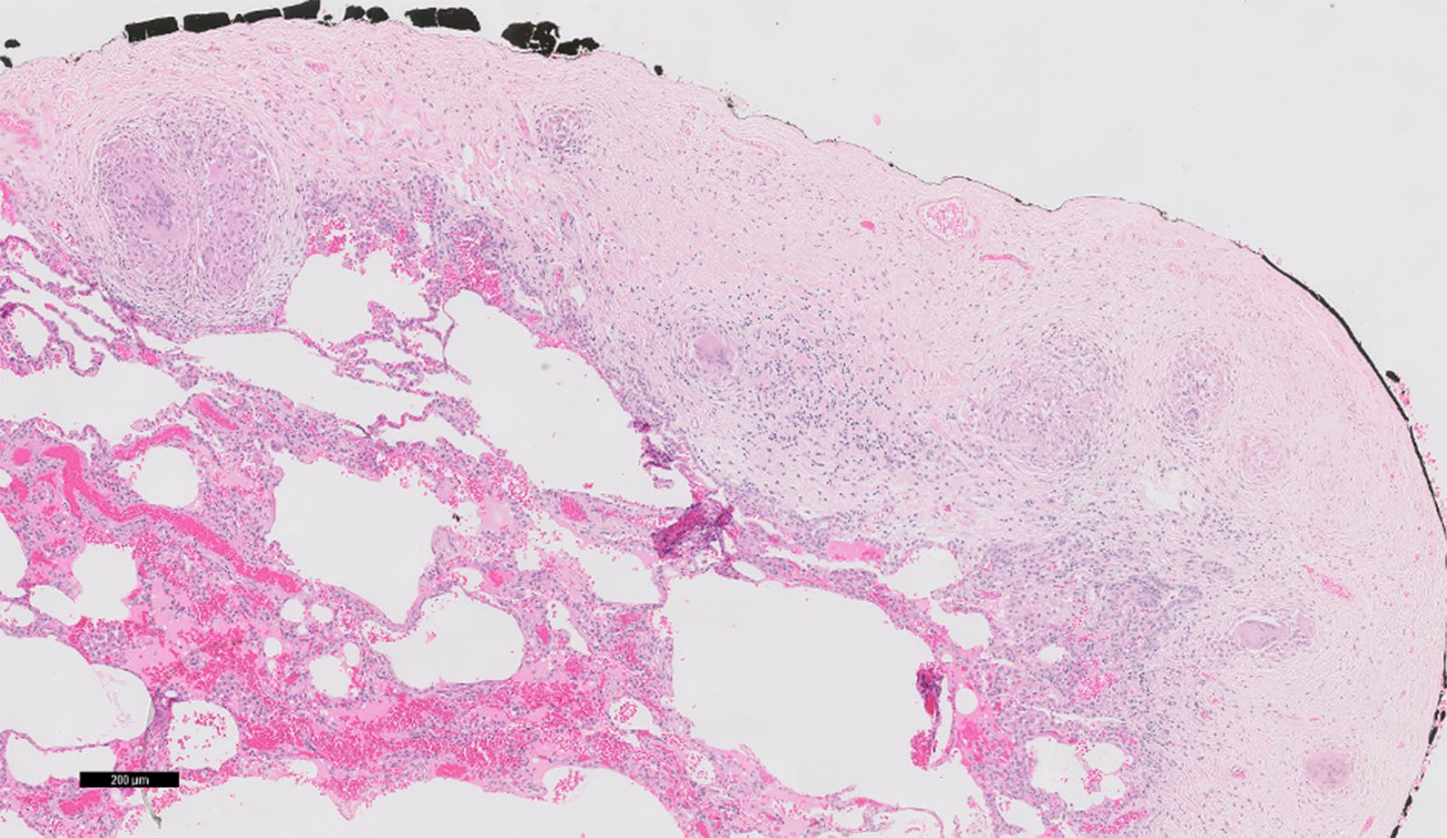
Video-assisted thoracoscopic biopsies of middle and right upper lobe showing pleural, interstitial non-necrotizing granulomas, compatible with sarcoidosis

Bronchoscopy with bronchoalveolar lavage showed lymphocytic inflammation; no mycobacterial, bacterial, fungal or viral pathogens were identified by culture or PCR. Video-assisted thoracoscopic biopsies of the right middle and upper lobes showed granulomas compatible with sarcoidosis but no other diagnostic clues (Fig. 3). Biochemical analysis of sputum showed nondiagnostic mucopolysaccharides; cultures remained negative.

Considering that her diagnosis—although with highly unusual presentation—best fit the earlier diagnosis of sarcoidosis [1, 4], we started her on infliximab [5]. We argued that TNF-α is the cytokine that plays a central role in the formation and maintenance of the granulomatous inflammatory response, even though most patients with pulmonary sarcoidosis benefit little from this treatment [6]. Infliximab is a chimeric, monoclonal immunoglobulin G1 (IgG1) antibody with dual effects: it neutralizes the effect of circulating TNF-α and resolves granulomas in affected tissues [7]. She received 4 mg/kg (200 mg) infliximab intravenously at 3- and later 6–12-week intervals and made a remarkable recovery; she resumed her part-time work as a teacher after a dropout of several years. On an attempt 2 years later to wean her from infliximab, she experienced a relapse, and after restarting three-monthly infliximab she has not experienced any relapses or intercurrent medical or surgical problems in subsequent years. At the time of writing this report, she was well.

## Discussion

This patient with sarcoidosis—radiographically, stage 1—presented with a highly unusual debilitating syndrome with cough productive of mucoid glazy material, without any evidence of infection, accompanied by fever and transient arthralgias.

Sarcoidosis patients typically have a dry cough [1]; production of sputum suggests an alternative diagnosis—mycobacterial infection, granulomatous airway involvement of Crohn’s disease [8] or diffuse panbronchiolitis [9]. Most patients with a productive cough typically also have bacterial organisms present in their sputum such as *Haemophilus influenzae* or *Pseudomonas* spp; in the 14 years we followed her, she never had bowel symptoms, making Crohn’s disease unlikely. She never had symptoms suggesting paranasal sinusitis, and her cough and sputum production subsided without macrolide use; these observations make the diagnosis of diffuse panbronchiolitis unlikely, and we therefore propose that all of her symptoms are consistent with a highly unusual presentation of sarcoidosis.

The workup included chemical and microbiological analysis of sputum and high-resolution CT, followed by bronchoscopic and video-assisted thoracoscopic biopsies, which were also cultured and subjected to PCR to detect a possible infectious origin. Taking all the evidence together, we conclude that infectious, metabolic, allergic, neoplastic and collagen-vascular disorders other than sarcoidosis could be ruled out.

We found one previously reported case of sarcoidosis presenting with a productive cough—but complicating bronchiectasis was not ruled out [10]. The sustained response to anti-TNF-α therapy during 12 years of follow-up suggests the latter [11]. As the incidence of sarcoidosis appears to increase over time, less common presentations might also become more prevalent [12].

## Conclusion

We describe a highly unusual presentation of sarcoidosis, with a cough productive of glazy mucoid sputum, accompanied by fever and transient arthralgias, radiographically stage 1, without evidence of complicating infection, and followed by complete resolution of symptoms with TNF-alpha inhibitor (infliximab) therapy, a response that persisted for over 12 years, and a relapse of symptoms following each of two different attempts to taper her infliximab therapy. This highly unusual presentation should be considered in patients with sarcoidosis and productive cough, after airway infection and bronchiectasis have been ruled out.

## Data Availability

Not applicable.

## Abbreviations

TNF-α: Tumor necrosis factor alpha
IgG1: Immunoglobulin G1
